# Clinical and Imaging Characteristics of Metastatic Orbital Tumours in North China

**DOI:** 10.1155/2024/3394425

**Published:** 2024-02-10

**Authors:** Yun Zhao, Shasha Yu, Mengxia Mu, Jiagen Li, Hongxun Li, Hong Zhao

**Affiliations:** ^1^Tianjin Eye Hospital, Tianjin Key Laboratory of Ophthalmology and Visual Science, Tianjin Eye Institute, Tianjin, China; ^2^Nankai University Affiliated Tianjin Eye Hospital, Nankai University, Tianjin, China; ^3^Clinical College of Ophthalmology (Tianjin Eye Hospital), Tianjin Medical University, Tianjin, China; ^4^Tianjin Occupational Diseases Precaution and Therapeutic Hospital (Tianjin Workers' Hospital), Tianjin, China

## Abstract

**Purpose:**

This study was designed to review the primary sites, clinical manifestations, imaging features, treatments, and outcomes of 36 patients with orbital metastasis in North China.

**Methods:**

This was a retrospective review of 36 patients with orbital metastasis at Tianjin Eye Hospital between January 2010 and December 2020 in North China as well as a review of the literature.

**Results:**

Thirty-six patients were included in the study; 17 were male, and 19 were female, with an age range of 1–82 years (average 54.9 ± 19.8 years). All the tumours were unilateral. The mean duration from the onset of orbital signs to presentation at the hospital was 2.4 months (range 1–10 months). Breast carcinoma, gastrointestinal tract carcinoma, and lung carcinoma were the most common histological types. Proptosis, ocular pain, and diplopia were the most common clinical manifestations. The superior orbit was the most common quadrant involved. All patients received comprehensive therapy, including surgery, radiotherapy, or chemotherapy. The average follow-up time was 2.45 years (range 7 months to 5.5 years). Ten patients in this study died as a result of disseminated metastasis from the primary tumour.

**Conclusions:**

In North China, the most common primary cancer that metastasizes to the orbit is breast cancer, followed by gastrointestinal tract carcinoma and lung cancer. The increasing trend of orbital gastrointestinal tract metastases in North China in recent years is noteworthy. The survival rate of patients with orbital metastasis of neuroblastoma is low.

## 1. Introduction

A number of tumours, inflammatory lesions, and congenital lesions can affect the orbit. According to the site of occurrence, orbital lesions can be classified as primary, secondary, or metastatic [1]. Orbital metastases are rare, and previous studies have reported that orbital metastases represent approximately 3∼7% of all orbital space-occupying lesions [2, 3]. Ophthalmologists play an important role in the diagnosis of orbital metastasis; 19% to 25% of patients may have no history of systemic cancer when presenting with ophthalmic symptoms [4]. Malignant tumours generally metastasize via the haematogenous route to the orbit [5]. Orbital metastases are less common than uveal metastases because of their blood supply [6]. The primary site of orbital metastasis can vary widely. Previous reports have suggested that prostate and lung carcinomas are common primary sites in males, whereas in females, breast carcinoma tops the list [7]. The clinical manifestations of orbital metastases vary. According to a case series of orbital metastases by Ahmad et al., diplopia, proptosis, pain, decreased vision, ptosis, and a palpable orbital mass were among the most common symptoms [8]. Most clinicians have little experience with orbital metastasis [6]. In the past, ophthalmologists could provide little treatment for patients with metastases, particularly those with orbital metastasis, which often heralds multiple organ involvement [9]. Owing to the lack of large sample size clinical studies from North China on orbital metastasis, we report 36 consecutive patients seen at Tianjin Eye Hospital between January 2010 and December 2020. In this study, we reviewed age, sex, symptoms, ophthalmic examinations, primary site and location, diagnosis, therapeutic methods, and prognosis. The English literature regarding orbital metastasis was also reviewed for further comprehension of this rare type of cancer.

## 2. Methods

All procedures involving human participants were performed in accordance with the ethical standards of the institutional and national research committee and with the 1964 Helsinki Declaration and its later amendments. The written informed consent form was signed by all patients after an explanation of the nature of the study. The study was approved by the Foundation Institutional Review Board of Tianjin Eye Hospital.

This retrospective study included 36 patients who were diagnosed with orbital metastasis at Tianjin Eye Hospital between January 2010 and December 2020. All patients were of Chinese ethnicity. All patients underwent surgical resection of the lesion, obtaining a gross total resection, followed by postoperative radiotherapy or chemotherapy.

Patients were not included in the study if there was evidence of direct orbital invasion from adjacent structures, and patients with lymphoproliferative disorders, such as lymphoma, leukaemia, or multiple myeloma, were excluded.

The main extracted data included sex, age, type of primary tumour, history of cancer, symptoms and duration, clinical manifestations, position, imaging findings, survival time, treatment, follow-up, and prognosis. All patients underwent ophthalmology examinations, computed tomography (CT), and histopathological examination. Most of the patients underwent magnetic resonance imaging (MRI), and some patients underwent fluorodeoxyglucose positron emission tomography/computed tomography (PET-CT). Only patients with evidence of metastatic cancer to the orbit via the haematogenous route were included.

## 3. Results

Thirty-six consecutive patients with orbital metastasis were identified and enrolled in this study. The mean age was 54.9 ± 19.8 years (range: 1–82 years), and the median age was 59 years. Of the 36 patients, 17 were males and 19 were females. Three patients (8.3%) were children. All patients were from the North provinces of China. All the tumours were unilateral (right: 17, left: 19) with no side predilection (*p* < 0.05), and no patients had bilateral involvement. The mean duration from the onset of orbital signs to presentation at the hospital was 2.4 months (range 1–10 months).

The most common orbital metastasis was breast carcinoma (9 patients, 25.0%), followed by gastrointestinal tract carcinoma (7 patients, 19.4%), lung carcinoma (5 patients, 13.9%), neuroblastoma (3 patients, 8.3%), liver carcinoma (2 patients, 5.6%), thyroid gland carcinoma (1 patient, 2.8%), prostate carcinoma (1 patient, 2.8%), kidney carcinoma (1 patient, 2.8%), uterine carcinoma (1 patient, 2.8%), malignant melanoma (1 patient, 2.8%), salivary gland carcinoma (1 patient, 2.8%), and others (4 patients, 11.1%). Breast carcinoma (9 patients, 100.0%) and uterine carcinoma (1 patient, 100.0%) affected only female patients. Gastrointestinal tract carcinoma (5 patients, 71.4%) and lung carcinoma (3 patients, 60.0%) predominantly affected males. There were only 3 children with orbital metastasis of neuroblastoma in our cohort. Most orbital metastases presented in patients with known primary cancer (31 patients, 86.1%). The other patients (5 patients, 13.9%) were confirmed to have orbital metastasis by PET-CT after surgery. The corresponding time interval was 1.4 months. Among the 5 patients, 2 had lung carcinoma, 1 had breast carcinoma, 1 had gastrointestinal tract carcinoma, and 1 had kidney carcinoma. The characteristics of the patients are shown in Table 1.

The presenting signs and symptoms of the 36 patients are listed in Table 2. Proptosis (91.7%), ocular pain (86.1%), diplopia (83.3%), limited ocular movement (80.6%), and chemosis (72.2%) were the five most common clinical manifestations, followed by ptosis (58.3%), visual loss (52.8%), eyelid swelling (50.0%), a palpable mass (33.3%), enophthalmos (8.3%), and others (8.3%). All 3 patients with enophthalmos had orbital metastasis of breast carcinoma.

**Figure 1:**
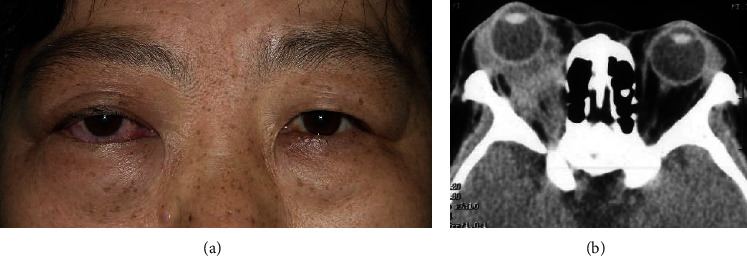
(a) Clinical photograph of a 49-year-old woman with orbital metastasis of breast cancer showed proptosis, ptosis, and chemosis of the right eye. (b) The coronal computed tomography showed an irregular mass in the right orbit, surrounding the eyeball, exophthalmos, with extraocular muscle involvement.

**Figure 2:**
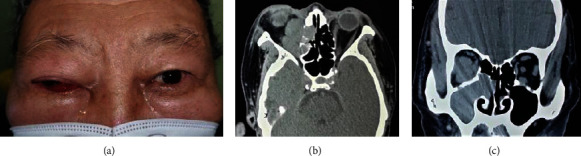
(a) Clinical photograph of a 76-year-old man with orbital metastasis of lung cancer showed ptosis, eyelid swelling, and chemosis of the right eye. (b) The coronal computed tomography showed a massive mass replacing the medial aspect of the orbit and ethmid sinus. (c) The coronal computed tomography scan showed an irregular mass in the right orbit replacing the orbit, ethmid sinus, and maxillary sinus.

**Figure 3:**
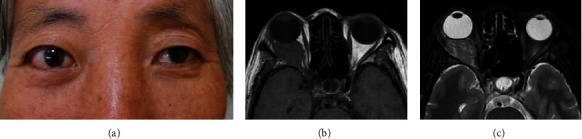
(a) Clinical photograph of a 49-year-old woman with orbital metastasis of gastrointestinal tract carcinoma showed proptosis, chemosis, and limited ocular movement of the right eye. (b) The transverse T1WI section showed that the tumour was isointense to the extraocular muscle and cerebral gray matter. (c) The transverse T2WI section showed minimal hyperintensity in the right orbit.

**Figure 4:**
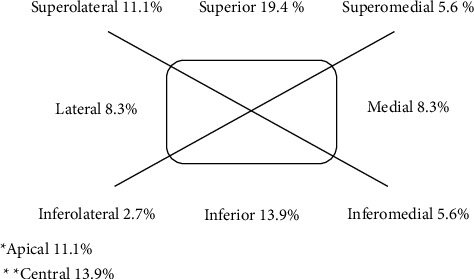
Localization of orbital metastasis.

Imaging data were available for all patients. Orbital CT scans were performed for all 36 patients. Twenty-three patients had a relatively high-density space-occupying soft tissue mass in the orbit involving the extraocular muscle without bony erosion (Figure 1). Thirteen patients had tumour invasion of the orbital wall, ethmoid sinus, maxillary sinus, or skull base (Figure 2). Twenty-one patients underwent MRI (Figure 3). Based on CT and MRI findings, the site of metastasis in the orbit could be determined. The most frequently invaded area was the superior orbit (19.4%), followed by the inferior orbit (13.9%), the central orbit (13.9%), the superolateral orbit (11.1%), and the apical orbit (11.1%) (Figure 4). Metastasis involved the extraocular muscle in 29 patients, the intraconal space in 15 patients, and the extraconal space in 21 patients.

All patients received comprehensive therapy, including surgery, radiotherapy, or chemotherapy. The specific treatment depended on the pathological type of tumour, the site of growth, and the purpose of treatment. All of the surgeries in our patients were performed by ophthalmologists via lateral orbitotomy or anterior orbitotomy. Among the 36 patients, 24 patients underwent complete resection, 6 patients underwent palliative resection, and 6 patients underwent biopsy for diagnostic purposes. Twenty-three patients (63.9%) received radiotherapy, and the median dose of radiation was 40 Gy (range, 10–70 Gy) delivered in average fractions of 2 Gy. Chemotherapy was used in 17 patients (47.2%) alone or in combination with other treatments.

**Figure 5:**
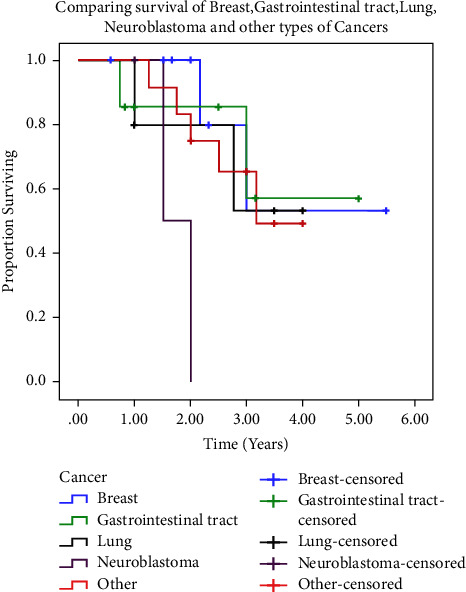
Survival curve of orbital metastasis. Kaplan–Meier curve with 95% confidence interval, showing gastrointestinal tract carcinoma having the best survival and neuroblastoma the worst.

Survival data were available for 35/36 patients. The average follow-up time was 2.45 years (range 7 months to 5.5 years). Ten patients in our study died as a result of disseminated metastasis from the primary tumour. Three patients died from unrelated causes. Overall survival differed significantly by cancer type (*p*=0.031), with gastrointestinal tract carcinoma having the best survival and neuroblastoma having the worst survival (Figure 5).

## 4. Discussion

Orbital metastases are relatively uncommon. The first documented case of orbital metastasis was a case of lung cancer metastasis to the orbit reported by Horner in 1864 [10]. Since then, several ophthalmologists have reported their experience with orbital metastases [11]. Several studies have shown that the incidence of orbital metastasis has increased over the past two decades compared to what was previously reported in the literature [12]. In the 21st century, the number of patients with systemic cancer with orbital metastasis has increased. The prevalence of orbital metastasis differs among hospitals, countries, and races. Yan and Gao reported their experience with orbital metastasis in South China [13]. However, there are no clinical studies on orbital metastasis in North China. Our hospital is a well-known tertiary ophthalmic centre in North China. According to previous reports, breast carcinoma, followed by lung carcinoma, is the most common source of orbital metastasis [14]. However, our study differed from previous studies.

Perhaps, the most interesting finding in our study of orbital metastasis, which distinguishes it from others, is the much greater incidence of metastatic gastrointestinal tract carcinoma. According to the literature, China has the largest number of incident cancer patients and deaths from liver cancer, oesophageal cancer, and gastric cancer, comprising 1.21 million (two-thirds of the world's total) newly diagnosed cases in 2020 [15]. These changes were mainly due to lifestyle changes in recent years. Therefore, the incidence of metastatic gastrointestinal tract carcinoma also increased with this trend. In contrast to a similar report in South China, there was no nasopharyngeal carcinoma in this cohort. This difference between North and South China is noteworthy. With the increasing incidence of gastrointestinal cancers in China, one must be vigilant for possible orbital metastasis in patients who present with diplopia, ptosis, eyelid swelling, and limited eye movement. In our study, there were slightly more females than males, but there were no significant sex-related differences. According to previous foreign reports, due to the large proportion of breast metastases, more female patients than male patients were reported. Among our patients, only two more had breast metastases than gastrointestinal metastases. Moreover, among the 7 patients with metastatic carcinomas of the gastrointestinal tract, 5 patients were male. Orbital metastasis is uncommon in childhood and occurs more often in adults. Among our patients, only 3 had orbital metastasis of neuroblastoma. Since our hospital is a specialized ophthalmic hospital, children with orbital metastasis of neuroblastoma were transferred to a children's hospital for comprehensive treatment after surgery.

Blood flow to the eye accounts for 2–5% of the total blood circulation. Thus, blood flow to the eye is very low compared to that to other organs [16]. Our study revealed that the most frequently invaded area was the superior orbit, followed by the inferior orbit, the central orbit, the superolateral orbit, and the apical orbit. The distribution of orbital metastasis may be related to the distribution of blood vessels in the orbit. Orbital metastasis was characterized by proptosis (91.7%), ocular pain (86.1%), diplopia (83.3%), limited ocular movement (80.6%) and chemosis (72.2%). However, it should be noted that orbital metastases located at the orbital apex may present as orbital apex syndrome or supraorbital tissue syndrome. Before surgery, we did not observe unilateral acquired blepharoptosis, which was reported in a previous study [17].

Imaging is very important for the diagnosis and treatment of orbital metastasis. The B-scan ultrasound and CT and MRI features of orbital metastases of different pathological types have been reported in previous literature. Orbital imaging via CT or MRI was helpful in guiding the biopsy to determine the appropriate surgical approach. Clinically, it is difficult to perform whole-body CT or MRI on patients, but PET-CT can provide more accurate information about orbital metastasis [18]. In recent years, the application of PET-CT in orbital metastasis has received increased attention [19]. PET-CT has been used as a new imaging method to improve the detection rate of orbital metastases [20]. In our series, 24 patients underwent PET-CT imaging. New metastatic lesions were found on PET-CT in 5 of these patients. PET-CT can help doctors stage orbital metastases and provide a reference for the design of treatment. Therefore, attention should be given to PET-CT images of patients with orbital metastasis before and after treatment.

Our study has inherent biases and limitations. All of the patients included in this study were from specialized ophthalmic hospitals, which limit the generalization of our results, possibly due to selection bias. We only collected patient data whose diagnosis was definitively confirmed through histopathological examination. Due to regional variability in our study and the fact that many patients were treated at other hospitals after surgery, these factors limit the accuracy of follow-up and the consistency of treatment protocols [21]. This study, to some extent, investigated the pathogenesis of orbital metastasis in North China, and the results of this study are quite different from those of previous studies in other areas.

In conclusion, our study revealed that breast cancer, gastrointestinal tract carcinoma, and lung cancer were the most common orbital metastases in North China. Orbital gastrointestinal tract metastases showed an increasing trend. Orbital metastatic neuroblastoma is mainly observed in children and has a poor prognosis. Orbital metastasis can display a variety of clinical and imaging features, although there is still a lack of uniform treatment.

## Figures and Tables

**Table 1:** Primary site, type of metastatic tumour, age, and sex in 36 patients.

Primary site	Tumour type	No. of patients	%	Mean age (range)	M	F
Breast	Carcinoma	9	25.0	56.4 ± 8.1 (47–71)	0	9
Gastrointestinal tract	Carcinoma	7	19.4	55.7 ± 11.3 (41–72)	5	2
Lung	Carcinoma	5	13.9	56.8 ± 20.4 (25–79)	3	2
Neuroblastoma	Carcinoma	3	8.3	2.3 ± 1.2 (1–3)	1	2
Liver	Carcinoma	2	5.6	59.5 ± 3.5 (57–62)	2	0
Thyroid gland	Carcinoma	1	2.8	69	0	1
Prostate	Carcinoma	1	2.8	74	1	0
Kidney	Carcinoma	1	2.8	66	1	0
Uterus	Carcinoma	1	2.8	71	0	1
Malignant melanoma	Carcinoma	1	2.8	82	1	0
Salivary gland	Carcinoma	1	2.8	49	1	0
Others	Carcinoma	4	11.1	68 ± 10.0 (54–77)	2	2
Total		36	100.0	54.9 ± 19.8 (1–82)	17	19

**Table 2:** The presenting symptoms and signs of 36 patients with orbital metastasis.

Symptoms and signs	No.	%
Proptosis	33	91.7
Ocular pain	31	86.1
Diplopia	30	83.3
Limited ocular movement	29	80.6
Chemosis	26	72.2
Ptosis	21	58.3
Visual loss	19	52.8
Eyelid swelling	18	50.0
Palpable mass	12	33.3
Enophthalmos	3	8.3
Other	3	8.3

## Data Availability

The data used to support the findings of this study are available from the corresponding author upon request.
